# What do patients think about HIV mass screening in France? A qualitative study

**DOI:** 10.1186/1471-2458-13-526

**Published:** 2013-05-30

**Authors:** Marie Paule Fernandez-Gerlinger, Erik Bernard, Olivier Saint-Lary

**Affiliations:** 1 Department of Infectious and Tropical Diseases, Bichat-Claude Bernard Hospital, Paris, France; 2 Department of General Medicine, Faculty of Health Sciences Simone Veil, University of Versailles Saint-Quentin-en-Yvelines, Montigny le Bretonneux, France

## Abstract

**Background:**

Since 2009, HIV mass screening of the 15–70-year-old general population in low-risk situations has been recommended in France. This, not yet implemented, untargeted screening would be cost-effective with a positive impact on public health. No previous studies had interrogated primary care patients about it. This study aimed at exploring perceptions of patients attending general practitioner’s on HIV mass screening and at identifying barriers to its implementation.

**Methods:**

We conducted a qualitative study through semi-structured individual interviews. Participants were recruited according to age, gender and location of their physician’s practice. Data analysis was based on triangulation by two researchers.

**Results:**

Twenty-four interviews were necessary to obtain data saturation. HIV transmission was mostly associated with sexual intercourse; main barriers stemming from the screening were related to sexuality, often seen as questioning spouse’s faithfulness. It could interfere with religiosity, implying an upsetting perception of sexuality among the elderly. Patients’ beliefs and perceptions regarding HIV/AIDS, the fear to be screened and difficulties to talk about sexuality were other barriers.

**Conclusion:**

To our knowledge, no studies had previously interrogated primary care patients about barriers to HIV mass screening in France. Although relevance of this untargeted screening is debated in France, our results could be helpful to a better understanding of patients’ attitudes toward this and to an outstanding contribution to reduce the number of new cases of HIV contamination.

## Background

About 33 million people are infected with the human immunodeficiency virus (HIV) worldwide [1]. In France, approximately 150,000 people are HIV-positive, among them 50,000 being unaware of it [2-4]. Between 6,000 and 8,000 new HIV-positive diagnoses are made each year, a third of these at an AIDS stage. In 2004, according to the French Institute for Public Health Surveillance (Institut de veille sanitaire, InVS), 47% of adults were not aware of being HIV-positive at the time of the diagnosis of AIDS, 46% of them were heterosexuals born in France [5]. In 2009, 62% of cases of infection resulted from heterosexual contact, and over 50% of urban cases occurred in Île-de-France [6].

Since 2009, mass screening of the low-risk 15–70-year-old general population has been recommended. This screening would be beneficial in terms of public health and cost-effective [7] although a recent French study questioned its utility [8]. Early diagnosis of HIV infection would have an individual and collective positive impact [9-14].

This untargeted screening in general population is not currently implemented in common practice and the 2009 recommendation has not made any change as the number of newly diagnosed HIV-positive cases has been stable since 2008 [15].

The use of qualitative methods is becoming more common in medical research in general and about HIV in particular [16,17] but few studies focused on the “patient” viewpoint [18]. The question of mass screening in general population has been mainly studied from a medico-economic perspective and in terms of public health impact [14,19-25]. Some qualitative studies have been conducted in the United States on the brakes and acceptance of HIV testing in primary care clinic in San Francisco [26] or querying only seropositive for HIV [27]. In the United Kingdom one study queried the feasibility of a rapid test in primary care [28]. But to our knowledge, no study has directly interviewed primary care patients in France about the opportunity of HIV mass screening.

Our study aimed at exploring perceptions of patients attending general practitioner’s on HIV mass screening and at identifying barriers to its implementation.

## Method

### Study type

A qualitative study through semi-structured individual interviews was performed [29,30]. Individual interviews were preferred to focus groups because the HIV theme is related to the private sphere and to sexuality and could compromise the quality of group exchanges [31]. We adhered to the RATS guidelines.

### Recruitment of participants

General practitioners exercising in Yvelines (one of the eight departments of the Île-de-France region, France) and internship supervisor at the Faculty of Health Sciences of Paris–Ile-de-France–Ouest invited their patients to take part in the study. The patients were recruited according to their age and gender, as well as the location of their physician’s practice, in order to obtain a great diversity. The participants should be aged 18 to 70 but we deliberately did not include minor patients (<18 year old) due to ethical issues and interview feasibility (a written agreement of both parents would have been required). A HIV-positive status already known by their attending physicians was an exclusion criterion.

The number of interviews was not set in advance. The objective was to achieve data saturation, defined in our study as the lack of any new theme raised during three consecutive interviews. Oral consent was sought systematically before each interview both by their attending GP and by the interviewer. The participants were advised that the interview could be interrupted at any time.

### Management of the interviews

The interviews were conducted by an intern of general medicine and took place at the practice of three general practitioners who were internship supervisors at the faculty of health sciences of Paris–Île-de-France–Ouest. Interviews took place in a quiet separate room, in order to respect patients’ privacy. An interview guide that was intentionally undetailed in order to facilitate expression (Appendix 1) was used to guide the interviews. Questions were deliberately open to limit potential bias linked to the researchers’ opinions on the subject [32]. Completion questions were provided in order to get more detailed answers but they were intentionally kept at a minimum. The participants were not incentivized.

### Data analysis

Several steps were followed to realize an inductive analysis of thematic content, with category construction based on analysis of the participants’ opinions. The inductive approach is a systematic procedure to analyze qualitative data in which the identified themes are a cluster of meaning like connected categories. Its primary purpose is to allow research findings to rise from the frequent, dominant or significant themes inherent in raw data. All the interviews were led by a resident in general practice, lasting from 3 to 15 minutes. Each individual interview was audio-recorded using two digital voice recorders, then transcribed into a computer file (Word). The recordings were destroyed after analysis out of respect for medical confidentiality and the private nature of the recorded data. The verbatim transcriptions were read several times, choosing units of meaning, identifying general themes, categorizing and classifying. The investigators’ triangulation involved two different researchers in the analysis process. The same method was used by each of them to analyze the data. Each evaluator’s findings were compared in order to optimize the data conformity.

### Ethical statement

The study has been reviewed by an ethics committee, the “*Comité de Protection des Personnes Île-de-France XI*” and the committee conclusions were that an ethical approval was not required under French law (law of the August 9, 2004).

## Results

In total, 24 interviews were needed to achieve data saturation. The participants (41-years-old in average) included 11 men and 13 women (Table 1).

**Table 1:** **Characteristics of participants (*n*** **= 24)**

**Patient**	**Age**	**Gender**	**Location of physician’s practice**
Patient 1	44	Female	Marly le Roi
Patient 2	37	Female	Marly le Roi
Patient 3	68	Male	Marly le Roi
Patient 4	62	Male	Marly le Roi
Patient 5	70	Male	Marly le Roi
Patient 6	47	Female	Marly le Roi
Patient 7	39	Male	Marly le Roi
Patient 8	25	Male	Versailles
Patient 9	34	Male	Versailles
Patient 10	20	Male	Versailles
Patient 11	49	Female	Versailles
Patient 12	33	Male	Versailles
Patient 13	53	Female	Versailles
Patient 14	19	Female	Versailles
Patient 15	51	Male	Rambouillet
Patient 16	20	Female	Rambouillet
Patient 17	53	Female	Rambouillet
Patient 18	54	Female	Rambouillet
Patient 19	31	Female	Rambouillet
Patient 20	69	Female	Rambouillet
Patient 21	40	Male	Rambouillet
Patient 22	40	Female	Rambouillet
Patient 23	21	Male	Versailles
Patient 24	18	Female	Versailles

### Barriers to the HIV mass screening related to sexuality

Most of interviewed patients associated HIV transmission with sexual relations, so they considered that the screening inevitably questioned their spouse’s fidelity: “*we cannot always have suspicions and get screened every 6 months”* (female patient no. 2). Marriage was seen by some as a guarantee of fidelity and therefore made the screening unnecessary: “*All married couples, if they’re faithful, I don’t think they need to be screened*” (male patient no. 9). Similarly, religion (Catholicism in our study) was supposed to protect patients from high-risk relations: “*young, very devout Catholics […] would only ever have sexual relations with their wife or husband […], they don’t even talk about HIV at home*” (female patient no. 24). Moreover, participants thought that sexuality was virtually inexistent among the elderly and questioned the need of screening for them: “*For old people, I think it’s not necessary […] I don’t think 70-year-old people have a sex life*” (female patient no. 2).

### Criticism of public policies

Several patients, particularly those older than 40, considered the cost of public health to be another barrier. “*Why incur those costs for the community?*” (female patient no. 6). Criticisms concerned public health policies and communication aimed at the general public: anticipated benefits of screening and lack of information about screening methods. Information overload was also criticized as it could lead to information saturation and induce distrust in prevention campaigns: “*Too much info kills the info. […] You get overloaded and you want to think about something else*” (female patient no. 13).

### HIV beliefs and perceptions

Fear of HIV-associated illness was considerable for some. Knowledge about this subject, even vague, only exacerbates this apprehension: “*some people wouldn’t dare to participate, because they’re afraid […]. This disease scares people*” (female patient no. 16). The fear of test result can add to that of the disease: “*They’re afraid to go find out the result*” (male patient no. 9).

The lack of screening of ‘others’ was viewed critically: “*I think they don’t think they have any chance of catching it, they feel well and have no symptoms, they don’t think they’re carriers*” (female patient 18). However, a concurrent feeling of being personally protected could exist: “*Not everyone is in danger of catching that disease […] From that point of view, I’m completely safe*” (male patient no. 4). Living in France could also provide a feeling of protection: “*It depends on the country. I don’t think France is much affected*” (male patient no. 8).

Patients also reported the embarrassment that sometimes prevents them from discussing about HIV, even with their physician: “*To be honest I thought of asking him, but I don’t really dare ask the doctor*” (female patient no. 19). “*It’s a taboo subject […] it’s like it’s something you shouldn’t talk about*” (male patient no. 23).

## Discussion

Our results provide a better insight into barriers to the HIV mass screening from the patients point of view and clarify their beliefs and perceptions.

The only mode of HIV transmission clearly identified by the interviewed patients was sexual contact. HIV was associated with unprotected sexual intercourses and infidelity, and patients were often unaware of the other modes of transmission, such as contact with blood or blood product (*e.g.* blood transfusion, intravenous drugs use, tattooing, piercings) and mother-to-child [33,34]. The fact that HIV transmission means sexual intercourse in patients’ mind, the HIV mass screening was susceptible to question fidelity within couple, to acknowledge the sexuality of the elderly and to raise discrepancies with religion. These factors could constitute barriers to the implementation of the screening.

The HIV mass screening could be perceived as questioning the fidelity between spouses and thus to be difficult to accept. How could patients agree to be screened for a disease against which the presumed fidelity of their spouse is supposed to protect them? The theme of fidelity (or infidelity) within relationships has been extensively studied in social sciences. According to studies conducted in Europe, Canada and the United States, near one third of men and one fifth of women have had relations outside of the couple [35]. Infidelity is found in all social classes in all countries, including the most repressive [35]. Despite these results, this widespread perception in patients was an obstacle to applying the recent French recommendations concerning HIV screening.

Patients believed that the screening was useless for patients over the age of 70, considering their sexuality as virtually non-existent or even disturbing. Although old age is associated with a decrease in desire and impulsivity, and sometimes with pain during the act [36], the sexuality of seniors is much more common than perceived in our sample. Sexual activity persisted into old age, sometimes with multiple partners [37]. In a study conducted in 1998 among a 80-102-year-old group, in residential retirement facilities, some had more than one partner [38]. Moreover, sex tourism of the elderly is also a factor in the transmission of sexual infections, for both men and women [39]. In 2003 and early 2004, the distribution of new cases of HIV and AIDS diagnosed in France, presented a rise over the age of 54, especially among men. From 2003 to 2006, nearly one out of six of all positive screening tests recorded were men over the age of 50 [40]. More recently, it has been estimated that by 2015, more than half of the patients seropositive for HIV in the United States would be more than 50 [41]; this population should be a new target of HIV screening [42]. Thus, even if age-related physiological changes can alter sexuality, the risk of infection by the AIDS virus remains well and truly real and should not be ignored by patients.

Some participants reported a religiosity that could be another barrier to systematic HIV screening. Catholicism is the most prevalent religion in France [43] and the Catholic Church promotes premarital chastity and is opposed to condom use [44] even if this is a more reliable method. Programs which only promote sexual abstinence are not effective in reducing the risk of HIV transmission [45-47]. Interviewed patients did not dwell on these controversies but mentioned that the Catholic religion could protect them against HIV if its rules were fully respected. This feeling of protection provided by religion could be another obstacle to screening acceptance.

### Limitation of the study

Our study took place in Yvelines, which is one of the most prosperous areas in France thus the patients we interviewed were not representative of the French population. However, the aim of this qualitative study was to get a better insight of the barriers coming from patients themselves towards the untargeted HIV screening, while avoiding potential subjective bias of the researchers. On the basis of our results, further quantitative studies can now be considered to quantify and rank the different factors according to their importance.

## Conclusion and recommendations

Systematic HIV screening in low-risk population is currently recommended by the French Health Authority [21] according to a favorable cost/effective ratio in recent studies [14]. Even if relevance of this screening is still debated [8,48], it seemed important to focus on the patients’ view, beyond the medico-economic considerations. Further HIV information campaigns could focus on the less frequent transmission modes in order to improve the general public awareness and change its perception about HIV mass screening. Talking about sexuality should be less taboo in the patient-doctor relationship and religious feeling could be more considered by practitioners when discussing with patients about HIV screening.

HIV mass screening implementation could reduce the number of new cases of HIV contamination and we think our results could facilitate patient-physician discussion regarding this screening.

Box 1: Summary of the barriers to HIV mass screening

HIV transmission was only associated with sexual relations

HIV screening inevitably questioned spouse’s fidelity

Religion (Catholicism) was supposed to protect patients from high-risk relations

Sexuality was supposed virtually inexistent among the elderly

Cost in Public Health

Lack of information about screening methods

Fear of HIV / fear of the result

Feeling of being personally protected, specially by living in France

## Appendix 1: Interview grid

1) The first for a “populational” angle, that of public health. The issue will be presented with the question: “***There are new recommendations to screen the whole general population for HIV, from the age of 15 to 70. Do you think the whole French population should be screened for HIV?***”. This will generate a consideration of the advantages and disadvantages of a health policy.

2) The second for a more “personal” angle:

“***Have you ever been screened for HIV?***”, to take stock.

•• If the answer is “yes”:

••was this following a high-risk situation?

“yes” → would you be screened outside of a high-risk situation?

“no” → what were your reasons?

••was it to know your status?

“yes” → what were your reasons?

“no” → under what circumstances?

••If the answer is “no”:

••would you only get screened in a high-risk situation?

“yes”: for what reasons?

“no”: for what reasons?

••would you get screened outside of a high-risk situation?

“yes”: for what purposes?

“no”: for what purposes?

3) The third, after information on the subject:

••Among the 150,000 people infected with HIV in France, 1/3 or 50,000 are unaware. According to you, what are the reason(s) for this figure?

••On a personal level, knowing these figures, would you get screened outside of a high-risk situation?

“yes”: why?

“no”: why?

## Competing interest

The authors declare that they have no competing interests.

## Authors’ contributions

MPF carried out the analysis and interpretation of data, EB participated in the design, revising it critically for important intellectual content, OSL participated in the design of the study, analysis and interpretation of data and final approval of the version to be published. All authors read and approved the final manuscript.

## Pre-publication history

The pre-publication history for this paper can be accessed here:

http://www.biomedcentral.com/1471-2458/13/526/prepub
